# Review of the Existing Evidence for Sex-Specific Relationships between Prenatal Phthalate Exposure and Children’s Neurodevelopment

**DOI:** 10.3390/ijerph182413013

**Published:** 2021-12-09

**Authors:** Agnieszka Jankowska, Linda Nazareth, Dorota Kaleta, Kinga Polanska

**Affiliations:** 1 Department of Environmental and Occupational Health Hazards, Nofer Institute of Occupational Medicine (NIOM), 91-348 Lodz, Poland; Kinga.Polanska@imp.lodz.pl; 2 Division of Students in English, Medical University of Lodz, 90-647 Lodz, Poland; Linda.Nazareth@stud.umed.lodz.pl; 3 Department of Hygiene and Epidemiology, Medical University of Lodz, 90-752 Lodz, Poland; dkaleta@op.pl

**Keywords:** sex-specific association, phthalate exposure, child neurodevelopment, behavior, cognition, psychomotor development

## Abstract

Phthalates are well-known, ubiquitous environmental contaminants influencing children’s health and their neurodevelopment. However, results of the previously conducted studies are not entirely conclusive. The aim of this review is to present the current state of knowledge with respect to the association between the prenatal phthalate exposure and sex-specific child neurodevelopmental outcomes. A systematic search of the literature was carried out to identify the studies that analyse the sex-specific association between prenatal exposure to phthalates and cognitive, psychomotor outcomes and behavioural and emotional problems. The search was conducted in May 2021, and it was limited to the papers published in English between January 2015 and April 2021. The following databases were used: PubMed, Scopus and Elsevier. The selection process was carried out by two independent authors according to the inclusion criteria. Of a total of 7542 records, 17 epidemiological studies met the inclusion criteria with regards to phthalate exposure and sex-specific differences in child neurobehavioural development. The review shows no clear pattern of association between maternal exposure to phthalates during pregnancy and offspring neurodevelopment. No clearly pronounced sex specific effects, except for BBzP exposure and decreased motor ablates among girls, have been indicated. Inconsistences in the results, as well as unsolved issues related to the interpretation of the results in the context of the exposure level, outcomes, confounders, and biological plausibility highlight the necessity for further research in the field.

## 1. Introduction

Child cognitive, psychomotor and behavioural development is determined by genetic as well as environmental and lifestyle-related factors, and by gen-environment interactions. Among the variety of environmental contaminants, phthalates are one the most frequently investigated ones. Phthalates are endocrine disrupting chemicals widely used as solvents, plasticizers, stabilizers, and additives in a number of products, including personal care products, food packaging, toys, building, constructions materials, and in medications [1]. General population is exposed to these chemicals via various exposure sources and routes with ingestion being the primary one [2,3]. Phthalates are rapidly metabolized in the body (with half-life of 3–18 h) and excreted in urine, which is a common, reliable, and non-invasive matrix for assessing phthalate exposure [4,5,6].

The existing up-to-date studies postulate that exposure to phthalates poses a health hazard with regard to reproductive function, non-communicable diseases including respiratory diseases, allergies, cardiometabolic diseases and neurodevelopmental disorders [7,8,9]. Furthermore, researchers stress that children experience higher exposure levels to most phthalates and consequently, present more pronounced health problems related to exposure in comparison to adults. This is due to their sensitive periods of exposure including prenatal and early childhood, their development, immaturity, diet, metabolism, and age-specific behaviours [10,11,12,13,14,15,16,17]. Phthalates cross the blood-placenta barrier [18] and thus, adversely affect the developing brain. Moreover, a continuous exposure in infancy and early childhood may contribute to long-term health consequences. The vast number of studies in this field suggest that exposure during these sensitive periods might be associated with behavioural, cognitive, and psychomotor consequences in children [14,19,20,21,22,23,24,25].

Results of the existing studies are not fully conclusive and consistent because of methodological discrepancies such as: study design, different exposure period (prenatal/infancy/childhood), phthalates and their metabolites considered, exposure level, use of a variety of tests for measuring neurodevelopmental outcomes and lack of appropriate control for confounding variables [14,19,20,21,22,23,24,26,27]. Additionally, some authors highlighted the importance of altered sex-specific differences in infant and child neurodevelopment [19,24,28]. There are several reviews summarizing the existing evidence on this topic [19,24,25,28,29,30]. Ejaredar et al., (2015), in a systematic review of the literature including studies published until year 2014, reported that boys were more prone to adverse behavioural and cognitive effects [19]. The second review covering 14 birth cohorts (papers published by May 2018) has pointed out that exposure to some phthalates had adverse effects on neurobehavioural development in children, with an increased likelihood of behavioural problems in boys [24]. Radke et al. (2020) based on the systematic review and meta-analysis (covering papers published by March 2019) concluded that overall, there is no clear pattern of association between prenatal phthalate exposure and child neurodevelopment, including cognition, motor effect and behaviour. Only motor effect of BBP in girls has been indicated with moderate evidence [30].

The action of phthalates is likely to involve several mechanisms. It is theorized that phthalates may perturb testosterone concentrations, thyroid hormones homeostasis, calcium signaling, lipid metabolism, steroid receptor activity, [27,31,32,33] and may disrupt dopaminergic neurons [34].

This review builds on the most recent studies regarding the association between prenatal phthalate exposure and children’s neurodevelopment with taking their sex into consideration. This review covers a notable number of new publications on this topic since the last review, which was published by Radke at al. (2020) [30]. Moreover, the number of evaluated phthalates/phthalate metabolites as well as neurodevelopmental outcomes is more extensive.

## 2. Materials and Methods

The PRISMA (preferred reporting items for systematic reviews and meta-analyses) was employed to guide this review [35].

A search of PubMed, Scopus, and Elsevier databases was performed in order to identify the studies which analyze the association between maternal exposure to phthalates during pregnancy and neurodevelopmental outcomes in children including their behaviour, cognitive and psychomotor development. The search was executed in May 2021. The search strategy was based on the following combinations of keywords: phthalate exposure, child exposure, child neurodevelopment, sex-specific behavior, cognition, psychomotor development.

The search was restricted to articles: (1) covering human studies; (2) published in English in peer reviewed journals since 2015; (3) evaluating phthalate metabolite concentrations (for 8 phthalates: di(2-ethylhexyl) phthalate (DEHP), di-n-octyl phthalate (DnOP), di-iso-nonyl phthalate (DiNP), di-methyl phthalate (DMP), di-ethyl phthalate (DEP), di-iso-butyl phthalate (DiBP), di-n-butyl phthalate (DnBP), and butyl-benzyl phthalate (BBzP) (Table S1) measured in maternal urine collected at least once during pregnancy; (4) assessing the following neurodevelopmental outcomes: (a) cognition (including general cognition, intelligence quotient (IQ), mental development index (MDI), language and memory abilities), (b) psychomotor skills (including summary scale of psychomotor developmental index (PDI) or fine and gross motor subscales) and (c) behaviour (including: externalizing and attention problems such as attention-deficit/hyperactivity disorder, oppositional-defiant disorder, and conduct disorder/problems; internalizing problems such as depression and anxiety), measured by means of accepted and validated tools; (5) presenting sex-specific relationships between prenatal phthalate exposure and children’s neurodevelopmental outcomes. The selection process was carried out independently by two authors according to the inclusion criteria. In the first step, the titles and abstracts were analyzed and then, the full text was reviewed. In total, 7542 articles were found, and they were all checked for eligibility. The majority of publications were excluded because of the following reasons: assessment of postnatal child exposure only, phthalate metabolite concentration measurements based on samples other than urine (e.g., breast milk, amniotic fluid, blood) and lack of sex-specific association between prenatal phthalate exposure and child neurodevelopment. Finally, 17 publications met eligibility criteria and have been included in the present review [36,37,38,39,40,41,42,43,44,45,46,47,48,49,50,51,52]. The number of publications (n = 17) does not represent the number of cohorts (n = 11) as in some cases there are several analyses and publications from the same study. From all the papers, the following information was extracted: study design and population (cohort name, study location, years of recruitment, sample size, mothers’ and children’s ages), timing of assessment of exposure, phthalates/phthalate’ metabolites and their concentrations, tools used for neurodevelopment assessment, confounders (Table S2) and the results of analyses. To assess the association between exposures and outcomes, the concept of defining the population, exposure, comparator, and outcomes (PECO) as described by Radke et al. (2020) was considered [30]. For the study evaluation we followed the methodology developed by Radke et al. (2020) (we have kept the already assigned assessment for the same studies and completed the assessment for the studies not included in the review by Radke at al., 2020) (Table S3). The details of the methodology have been provided elsewhere [30]. Briefly, the evaluation was performed for exposure, outcome, selection of the participants, confounding factors and analysis. For each domain it was possible to obtain a rating as: good, adequate, deficient, and critically deficient. Finally, based on the assessment given for each domain, an overall study classification was conducted with the following possible categories: high, medium, low or uninformative. This assessment was performed based on reviewer expert judgments (high was given to the studies that received good ranking for most domains, medium—to those which received adequate or good ranking across most domains, with the impact of any identified limitations not being judged as severe) [30]. Of 11 studies, 3 were classified as high and 7 as medium. One study was assessed as low (Kim et al. (2018)) and was not considered in the further analyses and interpretations [36].

## 3. Results

### 3.1. Characteristics of Studies

Table 1 presents characteristics of the epidemiological studies that met the inclusion criteria applied in the current review. The data included in the review came from 11 prospective cohorts conducted in America (6 studies), Asia (3 studies) and Europe (2 studies). The pregnant women who participated in the studies were between 16 and 46 years of age. The sample size of the included studies varied between 86 and 518 participants. Concentration of phthalate metabolites was determined in the urine samples according to the inclusion criteria. Table 2 shows concentrations of the phthalate metabolites in maternal urine collected during pregnancy or at delivery. Many of the studies analyzed the samples collected during the third trimester of pregnancy, while the data obtained from the HOME, INMA, CHAMACOS and Chinese cohorts was based on several time point assessments. The assessment of neurodevelopment was carried out with the participation of children aged from 27 weeks to 16 years. All the studies described above were adjusted for potential confounders; however, the number of confounding variables varied among the studies (Table S2). The following covariates were among the most frequently evaluated: child age, child sex, maternal age, socioeconomic status and marital status.

### 3.2. Prenatal Exposure to Phthalates and Child Cognition and Language Abilities

Table 3 presents the results of the studies analyzing the association between prenatal phthalate exposure and child cognition and language abilities. Four studies have evaluated the association between exposure to the sum of DEHP metabolites and child cognitive development. Only in the analysis performed by Hyland et al. (2019) sex-specific difference were observed, with a positive association among girls β: 1.6 (0.0, 3.3) and a negative one among boys β: −1.7 (−3.8, 0.3) (p for interaction = 0.01) [37]. Other studies do not show statistically significant and consistent results. When the DEHP metabolites were analyzed separately, there were not any statistically significant results for boys and for girls. Moreover sex-differences in the association were not noted. The impact of prenatal DEHP exposure on child language abilities was analysed in the study by Olsen et al. (2018), scored as of medium confidence [38]. That study indicated lower scores in language development with an increased prenatal phthalate exposure among boys and no associations among girls (with an opposite pattern observed).

Three studies present the association between prenatal BBzP exposure and child cognition; however, none of them indicates statistically significant associations neither in boys nor in girls. Some potentially sex specific associations between BBzP and neurocognitive development among boys and girls was postulated by Doherty et al. (2017) (*p* for interaction 0.05) and Qian et al. (2019) (*p* for interaction = 0.06); however, the patterns of associations are contradictory in those studies [39,40]. Ipapo et al. (2017) indicated sex specific associations between BBzP and visual recognition memory at the age of 27 weeks (*p* for interaction 0.01) [41].

There is no apparent consistency between the studies regarding the impact of prenatal DnBP exposure on child cognitive development. There is some suggestion of the effect modification by sex, so that girls may be more susceptible to cognitive effects of DnBP exposure than boys (girls: β: −2.78 (−5.03, −0.54); boys: β: 1.71 (0.08, 3.34); *p* for interaction 0.02) [39]. In the study by Ipapo et al. (2017) sex specific associations between DnBP and visual recognition memory at the age of 27 weeks was proposed (*p* for interaction 0.01) [41].

Among three studies analysing the association between prenatal MiBP exposure and child cognitive abilities, one medium confidence study has found sex-specific interaction with a significant inverse association observed in girls (girls: β: −2.28 (−4.33, −0.22), boys: β: 1.55 (−0.39, 3.48); *p* for interaction 0.01) [39].

Looking at the studies evaluating the impact of DEP exposure on child cognitive and language development the results are not consistent, with no sex-specific interaction presented.

Doherty et al. (2017) indicated a statistically significant negative association between prenatal MCPP exposure and child MDI among girls and an opposite pattern, although not statistically significant, among boys (*p* for interaction 0.007) [39]. There was no statistically significant association between prenatal exposure to DiNP and child language abilities in the study by Olsen et al. (2018) [38].

Summing up, the results for prenatal phthalate exposure and child cognition do not represent a significant pattern of association and sex-specific differences.

### 3.3. Prenatal Exposure to Phthalate and Child Psychomotor Development

Table 4 presents the results of the studies analyzing the association between prenatal phthalate exposure and child psychomotor development including fine and gross motor abilities. Four cohorts (one high and three medium confidence studies) present the sex specific analyses for the association between prenatal DEHP exposure (considered as the sum or individual metabolites) with one study presenting sex-specific differences of such an association (girls: β: −1.38 (−5.30, 2.54, boys: β: 3.24 (0.70, 5.78); *p* for interaction 0.05 [40]. However, no clears pattern of the association can be found based on the research.

Among four studies evaluating the impact of prenatal BBzP exposure on child motor functions three of them indicate a negative pattern in girls (with significant results reported by Doherty et al. (2017) and, Balalian et al. (2019)) [39,42] and 2 in boys (with one result being statistically significant (Balalian et al. (2019) [42]). Sex interaction was noted by Doherty et al. (2017) (*p* for interaction 0.04) [39].

In the analyses focusing of DnBP exposure and child motor development there was no clear sex specific pattern of association, with one study presenting a significant interaction with sex (girls: β: −2.29 (−4.63, 0.05), boys: β: 1.92 (0.31, 3.54); *p* for interaction 0.005) [39].

For prenatal DiBP exposure and child motor functions among both girls and boys a negative pattern of association, although not significant in all the analyses, except for general and gross functions among girls in Balalian et al. (2019) [42], was observed. No sex differences were noted.

Even though Balalian et al. (2019) [42] observed significant sex differences for the association between MEP exposure and general as well as gross motor functions (*p* for interaction 0.02 and 0.03 respectively) with the negative impact in girls, the other studies do not present consistent results [42].

For other phthalates and their metabolites, the results were based on single studies so a conclusion about their impact on child motor functions cannot be drown.

All in all, the existing studies indicate stronger negative associations between prenatal phthalate exposure, especially for BBzP, in girls, but additional evidence is needed.

### 3.4. Prenatal Exposure to Phthalate and Child Behavioural and Emotional Problems

Many analyses focus on the evaluation of the association between prenatal phthalate exposure and child behavioural and emotional outcomes, with a variety of dimensions being assessed (Table 5). Among them internalizing and externalizing problems are the ones which are most frequently analysed.

Seven analyses have focused on sex specific associations between prenatal exposure to DEHP or its specific metabolites and behavioural, and emotional outcomes in offspring. The results were statistically significant or consistent across the studies neither for internalizing nor for externalysing problems, with no sex specific differences noted.

Similarly, for the exposure to other phthalates (including DMP, DEP, DnBP, DiNP) the associations with behavioural and emotional problems were consistent neither in girls nor in boys. For DiBP, England-Mason et al. (2020) (assessed as a medium confidence study) presented a significant positive association with internalizing problems in boys; however, sex interaction was not observed (*p* for interaction 0.7) [43].

Summarizing, there is lack of consistency between the studies and the lack of sex differences for the impact of prenatal phthalate exposure on child behaviour.

In addition to the results presenting sex stratified analyses for the association between prenatal phthalate exposure and child neurodevelopmental outcomes, some studies only check if the effect estimate modification by sex exists. None of them indicates sex specific interaction [53,54,55,56,57].

## 4. Discussion

Data collected in this review shows no clear pattern of association between maternal exposure to phthalates during pregnancy and offspring neurodevelopment, with no clearly pronounced sex specific effects, except for BBzP exposure and decreased motor ablates among girls.

Such inconsistencies in the existing studies are also pointed out in the previously published reviews. A systematic review covering the studies published until 2014 has indicated the association between prenatal exposure to phthalates and adverse cognitive and behavioural outcomes in children, including lower IQ, attention problems and hyperactivity as well as poorer social communication [19]. Radke et al. have underlined that for each phthalate/outcome combination, there was slight or indeterminate evidence of an association, with the exception of motor effects for BBP, for which moderate evidence has been noted [30]. Minatoya and Kishi have concluded that prenatal exposure to phthalates may contribute to neurobehavioural outcomes in children, but the evidence is still limited [29]. Another review, the one by Palanza et al. (2021) underlined that animal and human studies demonstrate the evidence that EDCs exposure during critical periods of development affects sex differences in emotional and cognitive behaviours [28]. It has been also concluded that results are more heterogeneous when social, sexual, and parental behaviours are considered.

Differences in the results between the studies may be due to several reasons. Different phthalates metabolites were analyzed at various time periods, where most of studies collected one prenatal urinary sample during different trimesters of pregnancy. This might have resulted in an imprecise assessment of prenatal phthalate exposure because they have relatively short biological half-lives. Moreover, some phthalates are relatively structurally similar and moderately correlated with one another (e.g., DBP and DIBP), while others differ considerably in terms of structure, sources of exposure as well as represent low correlations (DEP and DEHP) [30]. In addition, concentrations of phthalate metabolites varied between the countries. Additionally, different tools were used to assess children’s cognitive, psychomotor, behavioural, and emotional development and different covariates were considered in the performed analyses.

The proposed mechanism linking prenatal phthalate exposure to the adverse effects on children’s neurobehavioural development although still not fully established, assumes that phthalates can have an adverse effect on the systems and processes that are crucial for the development of the fetal brain and nervous system [27,58]. Phthalates and their metabolites can interact with sex-hormones, either in the pregnant woman or in the developing fetus [59,60]. The rodent studies have pointed out that the females are more emotionally vulnerable, while males are more cognitively susceptible, specifically in spatial memory [61,62].

It is established that action of certain metabolites of phthalates can result in an abnormal production and functioning of specific thyroid hormones [31,63,64,65]. Thyroid hormone homeostasis is crucial for fetal and infant brain development and in consequence may impact children’s cognitive and motor abilities [27]. Morgenstern et al., (2017) reported inverse and sex-specific associations between some metabolites of phthalates determined in early childhood and thyroid function in preschool children [66]. These results support the assumption that lower thyroid hormones mediate the association between exposure to phthalates in early life and child cognitive outcomes. Furthermore, phthalates may disrupt dopaminergic neurons [34], which are associated with the progression of motor skills, calcium signaling and lipid metabolism, all of which play important roles in various neurodevelopment processes [67]. Moreover, phthalate environmental exposure has a considerable influence on the brain. This fact is invariably supported by data that presents damage to the hippocampal structural and functional plasticity [68]. The hippocampus has a prominent role in learning and memory [69]. In one study, developmental exposure to certain phthalates has resulted in a reduced number of neurons and synapses in the medial prefrontal cortex [70].

The consequences of maternal exposure to phthalates for child development depend on both genetic and environmental factors, as well as their interactions, which determine individual susceptibility to their effects. Thus, identification of such factors and their influence on the occurrence of children’s neurobehavioural and neurodevelopmental disorders is of high scientific, public health and clinical relevance, consequently making it a priority public health target. Further prospective studies are required to consider sex as a statistical variable and to elucidate sex differences in susceptibility with respect to environmental factors that children are exposed to.

## 5. Conclusions

The results exhibited in this review are not entirely conclusive, with the sex specific effect not clearly pronounced. The considerable limitations in the existing literature, which are mostly related to misclassification in the exposure assessment, different tools used to assess children’s neurodevelopment and different covariates considered in the performed analyses need to be highlighted. Studies addressing those limitations are crucial and might help to understand which phthalates are important factors determining child cognitive, psychomotor, behavioural and emotional development. Such knowledge can be translated into effective public health actions to protect this vulnerable population.

## Figures and Tables

**Table 1:** Characteristics of studies included in the systematic review.

References	Name of Study	Location	Years of Recruitment	Age of Pregnant Woman (in Years)	Sample Size	Exposure Assessment	Children’s Age at Neurodevelopment Assessment	Tools Used for Neurodevelopment Assessment
Gascon et al. (2015)	INMA: INfancia y Medio Ambiente	Catalonia (Spain)	2004–2006	≥ 16	367	Two urine samples (12 and 32 wk gest) Σ4DEHP (MEHP, MEHHP; MEOHP; MECPP), MBzP, MEP, MiBP, MnBP	1, 4 and 7 years	BSID II—at age 1; MSCA, CPSCS, ADHD-DSM-IV—at age 4; SDQ, CSRS—at age 7 years
Lien et al. (2015)	NTMIC: The nationwide Taiwan Maternal and Infant Cohort	Taiwan	2000–2001	25–35	122	Single urine sample (28–36 wk gest) MEHP, MEHHP, MEOHP, MMP, MEP, MBP, MBzP	8–9 years	CBCL
Huang et al. (2019)					153		8–9, 11–12, 14–15 years	CBCL
Percy et al. (2016)	HOME: The Health Outcomes and Measures of the Environment	Cincinnati (USA)	2003–2006	≥18	227	Two urine samples (16 and 26 wk gest) Σ4DEHP (MEHP, MEHHP; MEOHP; MECPP), MEP, MnBP, MBzP, MiBP, MCPP	8 years	GIQ, PPPSI
Braun et al. (2017)					198		8 years	VMWM
Li et al. (2020)					314		2, 3, 4, 5 and 8 years	BASC-2
Ipapo et al. (2017)	CCCEH: The Columbia Center for Children’s Environmental Health	New York City (USA)	1999–2006	18–35	168	Single urine sample (34 wk gest) Σ4DEHP (MEHP, MEHHP, MEOHP, MECPP), MiBP, MBzP, MnBP, MCPP, MEP	27 weeks	FTII
Balalian et al. (2019)					209	Single urine sample (34 wk gest) Σ4DEHP (MEHP, MEHHP; MEOHP; MECPP), MiBP, MBzP, MnBP, MCOP, MEP	11 years	BOT-2
Daniel et al. (2020)						Single urine sample (34 wk gest) DEHP (MEHHP, MEOHP, MECPP), Non-DEHP (MBP, MEP, MiBP, MBzP)	11 years	BOT-2
Daniel et al. (2020b)					322	Single urine sample (34 wk gest) Σ4DEHP (MEHP, MEHHP; MEOHP; MECPP), MiBP, MBzP, MnBP, MEP	7 years	CPRS, CBCL
Doherty et al. (2017)	MSCEHC: The Mount Sinai Children’s Environmental Health Center	New York City (USA)	1998–2002	Me = 22	258	Single urine sample (25–40 wk gest) Σ4DEHP (MEHP, MEHHP; MEOHP; MECPP), MEP, MnBP, MiBP, MCPP, MBzP	2 years	BSID-II
Kim et al. (2018)	CHECK: Children’s Health and Environmental Chemicals in Korea	Seoul, Anyang, Ansan, Jeju (South Korea)	2011–2012	23–46	86	Single urine sample (at delivery) MEHP, MEHHP, MEOHP, MiBP, MnBP, MEP	13–24 months	BSID-II; SMS; CBCL
Olesen et al. (2018)	OCC: Odense Child Cohort	Denmark	2010–2014	No data	Vocabulary: 518, Complexity: 384	Single urine sample (28 wk gest) Σ4DEHP (MEHP, MEHHP, MEOHP, MECPP), Σ3DiNP (MhiNP, MCiOP, MOiNP) Σ2DBP (MiBP, MnBP), MEP, MBzP	20–36 months	MB-CDI
Hyland et al. (2019)	CHAMACOS: Center for the Health Assessment of Mothers and Children of Salinas	California’s Salinas Valley (USA)	1999–2000	≥18	334	Two urine samples (13 and 26 wk gest) Σ4DEHP (MEHP, MEHHP, MEOHP, MECPP), MEP, MBP, MiBP, MBzP, MCOP, MCNP, MCPP	7–16 years	WISC–IV—at age 7–10, 5 y; ENI—at age 9 y; NEPSYII — at age 12 y; SRS—2 at age 14
Qian et al. (2019)	Prenatal cohort in Wuhan, China	Wuhan (China)	2014–2015	28.6 ± 3.4	476	Multiple urine samples (the 1st, 2nd, and 3rd trimester) Σ4DEHP (MEHP, MEHHP, MEOHP, MECPP), Σ2DBP (MiBP, MnBP), MEP, MBzP	23–26 months	BSID
England—Mason et al. (2020)	APrON: Alberta Pregnancy Outcomes and Nutrition (Cohort)	Alberta, (Canada)	2009–2012	25–38	351	Single urine sample (17 wk gest) MEHP, MEHHP, MEOHP, MECPP, MBP, MiBP, MBzP, MEP, MMP	3–4 years	CBCL, BASC-2
Oulhote et al. (2020)	MIREC: The Maternal–Infant Research on Environmental Chemicals (Cohort)	ten cities in Canada	2008–2011	>18	510	Single urine sample (6–14 wk gest) Σ3DEHP (MEHP, MEHHP, MEOHP), MEP, MBP, MBzP, MCPP	3–4 years	SRS-2

Scale abbreviations: ADHA-DSM-IV: Attention Deficit Hyperactivity Disorder (ADHD) Criteria of the Diagnostic and Statistical Manual of Mental Disorders—4th Edition; BASC-2: Behavioural Assessment System for Children-2; BSID: Bayley Scales of Infant and Toddler Development; BSID-II: Bayley Scales of Infant Development- II; BOT-2 Bruininks—Oseretsky Test of Motor Proficiency—2nd edition; CBCL: Child Behavior Checklist; CPSCS: California Preschool Social Competence Scale; CSRS: The Conners’ Parent Rating Scales; CPRS: The Conners’ Parent Rating Scale-Revised: Long Form; ENI: Evaluación neuropsicológica del niño; FTII: The Fagan Test of Infant Intelligence; GIQ: Gender Identity Questionnaire; MB-CDI: The MacArthurBates Communicative Development Inventories; MSCA: McCarthy Scales of Children’s Abilities; NEPSY-II Affect Recognition subtest; PPPSI: Playmate and play style preferences structured Interview; SRS-2 Social Responsiveness Scale—Version 2; SDQ: the Strengths and Difficulties Questionnaire; SMS: Social Maturity Scale; WPPSI-III: Wechsler Preschool and Primary Scale of Intelligence; WISC—IV Wechsler Intelligence Scale for Children—4th edition; VMWM: the Virtual Morris Water Maze.

**Table 2:** Concentrations of the phthalate metabolite in maternal urine (µg/L, µg/g creatinine).

Reference	Gascon et al. (2015)	Lien et al. (2015)	Huang et al. (2019)	Percy et al. (2016)		Braun et al. (2017)	Li et al. (2020)	Ipapo et al. (2017)	Balalian et al. (2019)	Daniel et al. (2020b)	Doherty et al. (2017)	Kim et al. (2018)	Olesen et al. (2018)	Hyland et al. (2019)	Qian et al. (2019)	England-Mason et al. (2020)	Oulhote et al. (2020)
Exposure timing during pregnancy	12 and 32 wk gest	28–36 wk gest	28–36 wk gest	16 wk gest	26 wk gest	16 and 26 wk gest	16 and 26 wk gest	34 wk gest	34 wk gest	34 wk gest	25–40 wk gest	Single urine sample	28 wk gest	13 and 26 wk gest	1st, 2nd, 3rd Trimesters ^f^	17 wk gest	6–14 wk gest
Phthalates/ phthalate metabolites	Median (µg/g creat.)	GM (95% CI) (µg/g creat)	GM (95% CI) (µg/g creat)	GM (95% CI) (µg/L)		Median (µg/g creat)	Median ^@^	GM (95% CI) (µg/L)	GM (95% CI) (µg/L)	GM (95% CI) (µg/L)	GM (µg/L)	Median (IQR) ^b^ (µg/g creat)	GM (95% CI) (µg/L)	GM (GSD) (µg/L)	Median (µg/L)	GM (µg/L)	GM (IQR) ^b^ (µg/L)
ΣDEHP	99.8			87.9 * (73.4–105.3)	65.9 * (55.2–78.5)	69	1.87		281.9 (241.4–329.4) ^e^	294.7 (260.0–334.0)	0.28 ▪		17.5 ^◊^ (16.0–19.2)	0.2 (2.2)	91.71 ^c^		18.1 (12.0–25.4)
ΣDBP															407.35 ^d^		
MEHP	10.7	16.93 (14.32–20.02)	16.73 (14.46–19.36)	4.9 (4.1–6.0)	4.3 (3.6–5.0)			4.70 (3.91–5.65)	4.8 (3.7–5.4)	5.2 (4.5–6.0)	6.2	12.8 (7.8–19.1)	1.2 (1.1–1.3)	4.5 (2.6)	3.23	3.31	
MEHHP	27.1	7.91 (5.69–11.02)	7.07 (5.19–9.64)	27 (22.3–32.7)	19.4 (16.1–23.4)			19.99 (16.81–23.78)	21.2 (17.9–25.1)	22.1 (19.3–25.4)	20	25.3 (14.9–35.4)	4.7 (4.4–5.1)	18.9 (2.4)	6.91	10.38	
MEOHP	20.6	13.59 (10.27–18.00)	12.79 (9.88–16.57)	20.1 (16.7–24.2)	15.9 (13.2–19.2)			16.44 (13.83–19.56)	17.6 (14.4–20.8)	18.4 (16.1–21.0)	18	22.3 (13.5–31.1)	3.9 (3.6–4.3)	13.8 (2.4)	5.62	8.45	
MECPP	39.0			39.3 (33–46.9)	29.1 (24.5–34.6)			34.07 (29.15–39.81)	37.7 (32.5–43.7)	39.1 (34.7–44.1)	35		4.6 (4.2–5.0)	32.4 (2.2)	10.62	14.81	
MCPP				2.4 (2.1–2.8)	1.6 (1.4–1.9)	2.2	0.35	1.96 (1.68–2.29) ^a^		2.0 (1.8–2.2)				2.2 (2.3)			0.98 (0.49–1.76)
MBzP	11.9	15.88 (13.87–18.19)	14.99 (13.23–16.98)	9.3 (7.7–11.3)	7.7 (6.2–9.4)	9.5	0.97	12.23 (10.04–14.89)	12.3 (10.3–14.8)	13.3 (11.5–15.4	14		3.7 (3.4–4.1)	8.9 (2.6)	2.46	8.13	5.4 (3.1–9.1)
MiBP	31.6			4.8 (4.1–5.7)	3.7 (3.1–4.4)	5.2	0.69	6.88 (5.82–8.12)	7.5 (6.5–8.7)	9.1 (8.1–10.2)	5.6	7.6 (4.8–18.4)	24.3 (22.2–26.6)	3.4 (2.7)	19.50	9.71	
MnBP	30.8			24 (20.7–27.7)	20.6 (17.3–24.5)	24	1.39	33.45 (28.29–39.55)	35.7 (30.8–41.4)	37.4 (33.3–42.0)	33	41.0 (30.0–50.8)	12.4 (11.4–13.5)		68.10		
MMP		54.53 (44.95–66.14)	52.38 (44.0–62.36)														
MEP	403.4	61.71 (52.45–72.60)	63.70 (55.07–73.67)	141.8 (118–170.4)	115.3 (92.7–143.3)	128	2.11	165.69 (137.4–199.8)	148.7 (126.6–174.7)	160.4 (140.4–183.2)	200	11.9 (4.0–26.1)	22.0 (19.4–24.9)	241.7 (3.3)	11.29	50.41	33.1 (12.4–70.3)
MBP		66.88 (55.93–79.99)	67.29 (57.78–78.38)											28.4 (2.4)		16.66	12.8 (7.9–19.2)
MCOP									2.1 (1.7–2.7)					3.8 (2.1)			
MCNP														2.3 (2.0)			
MiNP													1.0 (0.8–1.3)				
MHiNP													1.8 (1.7–2.0)				
MOiNP													1.4 (1.2–1.5)				
MCiOP													3.6 (3.3–4.0)				

* ΣDEHP expressed as MEHP, ng/mL (=molar sum of (MECPP, MEOHP, MEOHP, MEHP) in nmol/L); ^◊^ ΣDEHPm molar sum of DEHP metabolites expressed as excreted DEHP (ng/mL); ▪ DEHP µmol/L; ^a^ MCPP is major metabolite of DnOP, secondary metabolite of DnBP and a non-specific metabolite of various high molecular weight phthalates; ^b^ Interquartile range (IQR) showing the 25th and 75th percentile values; ^c^ ΣDEHP: the sum of molar concentrations (nmol/L) of MEHP, MEHHP, MEOHP and MEOHP; ^d^ ΣDBP: the sum of molar concentrations (nmol/L) of MnBP and MiBP; ^e^ Σ DEHP is the total molar sum of: MEHP, MEHHP, MECPP and MEOHP; ^f^ averaged concentrations during pregnancy; ^@^ Urinary phthalate metabolite values (ng/mL) were divided by creatinine concentrations (mg/dL) and multiplied by 100 and then log10-transformed, measurement-error-corrected values were presented; GM—geometric mean. GSD—geometric standard deviation. Daniel et al. (2020) study was conducted on the data from an earlier study, Balalian et al. (2019).

**Table 3:** Sex-specific difference between phthalates exposure and child cognition.

Phthalates/ Phthalate Metabolites	References/Study Confidence	Outcomes/Years	Child Cognitive Development Effects β (95%CI), OR (95%CI)		*p*_interaction_
			Females	Males	
Σ4DEHP					
	Gascon ^a^ et al. (2015); high	Cognition (1 year)	β: 0.2 (−3.4, 3.7)	β: 0.3 (−3.1, 3.7)	
		Cognition (4 years)	β: 1.3 (−1.7, 4.3)	β: 2.8 (−0.6, 6.2)	
	Ipapo et al. (2017); medium	Visual recognition memory (27 weeks)	β: −4.23 (−8.13, −0.32)	β:−1.61 (−5.05, 1.82)	0.59
	Hyland et al. (2019); high	FSIQ—Full-Scale IQ (7, 10.5 years)	β: 1.6 (0.0, 3.3)	β: −1.7 (−3.8, 0.3)	0.01
	Doherty et al. (2017); medium	Mental Development Index (2 years)	β: 1.77 (−0.13, 3.67)	β: 0.10 (−1.48, 1.68)	0.2
	Qian et al. (2019); medium	Mental Development Index (2 years)	β: −0.89 (−5.13, 3.35)	β: −1.45 (−4.73, 1.83)	0.84
	Olesen * et al. (2018); medium	Vocabulary scores (20–36 months)	OR: 0.82 (0.61, 1.11)	OR: 1.33 (1.01, 1.75)	
		Language complexity scores (20–36 months)	OR: 0.88 (0.65, 1.18)	OR: 1.43 (1.03, 1.97)	
MEHP					
	Ipapo et al. (2017); medium	Visual recognition memory (27 weeks)	β: −3.05 (−6.75, 0.66)	β: 0.35 (−3.05, 3.75)	0.42
	Qian et al. (2019); medium	Mental Development Index (2 years)	β: −1.54 (−3.61, 0.53)	β: −0.44 (−2.65, 1.77)	0.48
	Olesen et al. (2018); medium	Vocabulary scores (20–36 months)	OR: 0.88 (0.71,1.09)	OR: 1.02 (0.85, 1.23)	
		Language complexity scores (20–36 months)	OR: 1.08 (0.86,1.35)	OR: 1.14 (0.92, 1.42)	
MEHHP					
	Ipapo et al. (2017); medium	Visual recognition memory (27 weeks)	β: −2.92 (−6.81, 0.10)	β: −4.25 (−7.67, −0.82)	0.57
	Olesen et al. (2018); medium	Vocabulary scores (20–36 months)	OR: 0.87 (0.67, 1.13)	OR: 1.32 (1.03, 1.70)	
		Language omplexity scores (20–36 months)	OR: 0.86 (0.66, 1.11)	OR: 1.38 (1.04, 1.83)	
	Qian et al. (2019); medium	Mental Development Index (2 years)	β: −0.73 (−4.89, 3.42)	β: −1.27 (−4.54, 2.01)	0.85
MEOHP					
	Ipapo et al. (2017); medium	Visual recognition memory (27 weeks)	β: −3.09 (−6.85, 0.68)	β: −2.27 (−5.79, 1.25)	0.82
	Olesen et al. (2018); medium	Vocabulary scores (20–36 months)	OR: 0.81 (0.63, 1.05)	OR: 1.35 (1.04, 1.73)	
		Language complexity scores (20–36 months)	OR: 0.89 (0.68, 1.16)	OR: 1.41 (1.06, 1.87)	
	Qian et al. (2019); medium	Mental Development Index (2 years)	β: 0.07 (−4.22, 4.35)	β: −2.25 (−5.46, 0.95)	0.42
MECPP					
	Ipapo et al. (2017); medium	Visual recognition memory (27 weeks)	β: −2.65 (−6.36, 1.06)	β: −0.09 (−3.58, 3.40)	0.12
	Olesen et al. (2018); medium	Vocabulary scores (20–36 months)	OR: 0.83 (0.62, 1.11)	OR: 1.37 (1.02, 1.85)	
		Language complexity scores (20–36 months)	OR: 0.90 (0.67, 1.21)	OR: 1.48 (1.04, 2.09)	
	Qian et al. (2019); medium	Mental Development Index (2 years)	β: −0.53 (−4.72, 3.66)	β: −2.59 (−5.95, 0.77)	0.47
MBzP					
	Gascon ^a^ et al. (2015); high	Cognition (1 year)	β: 1.0 (−1.9, 4.0)	β: 0.4 (−2.1, 2.8)	
		Cognition (4 years)	β: −0.5 (−3.1, 2.1)	β: 1.1 (−1.3, 3.5)	
	Ipapo et al. (2017); medium	Visual recognition memory (27 weeks)	β:−3.98 (−7.71, −0.25)	β: 2.39 (−1.26, 6.04)	0.01
	Doherty et al. (2017); medium	Mental Development Index (2 years)	β: −0.58 (−2.25, 1.09)	β: 1.83 (0.07, 3.58)	0.05
	Olesen et al. (2018); medium	Vocabulary scores (20–36 months)	OR: 0.93 (0.74, 1.18)	OR: 1.08 (0.89,1.32)	
		Language complexity scores (20–36 months)	OR: 0.96 (0.76, 1.21)	OR: 1.28 (1.02, 1.59)	
	Qian et al. (2019); medium	Mental Development Index (2 years)	β: 1.17 (−0.46, 2.80)	β: −1.23 (−3.12, 0.66)	0.06
ΣDBP					
	Qian et al. (2019); medium	Mental Development Index (2 years)	β: −0.32 (−3.16, 2.52)	β: −0.16 (−3.22, 2.91)	0.94
	Olesen et al. (2018); medium	Vocabulary scores (20–36 months)	OR: 0.81 (0.61, 1.08)	OR: 1.01 (0.77, 1.32)	
		Language complexity scores (20–36 months)	OR: 0.97 (0.74, 1.27)	OR: 1.12 (0.81, 1.54)	
MnBP					
	Gascon ^a^ et al. (2015); high	Cognition (1 year)	β: 1.4 (−1.5, 4.2)	β: 1.5 (−1.6, 4.6)	
		Cognition (4 years)	β: 0.4 (−2.0, 2.8)	β: 1.7 (−1.4, 4.7)	
	Braun et al. (2017); high	Visual-spatial abilities (8 years)	β: −1.7 (−2.8, −0.5)	β: −3.0 (−5.6, −0,4)	
	Doherty et al. (2017); medium	Mental Development Index (2 years)	β: −2.78 (−5.03, −0.54)	β: 1.71 (0.08, 3.34)	0.002
	Ipapo et al. (2017); medium	Visual recognition memory (27 weeks)	β: −2.40 (−6.13, 1.32)	β: 3.32 (−043, 7.08)	0.01
	Olesen et al. (2018); medium	Vocabulary scores (20–36 months)	OR: 0.81 (0.63,1.05	OR: 1.11 (0.86, 1.42)	
		Language complexity scores (20–36 months)	OR: 0.89 (0.69, 1.16)	OR: 1.31 (0.97, 1.77)	
	Qian et al. (2019); medium	Mental Development Index (2 years)	β: −0.33 (−2.91, 2.25)	β: −0.28 (−2.92, 2.35)	0.98
MiBP					
	Ipapo et al. (2017); medium	Visual recognition memory (27 weeks)	β: −3.12 (−6.66, 0.42)	β: −1.16 (−4.71, 2.39)	0.35
	Olesen et al. (2018); medium	Vocabulary scores (20–36 months)	OR: 0.86 (0.65, 1.13)	OR: 0.99 (0.76, 1.30)	
		Language complexity scores (20–36 months)	OR: 1.01 (0.77, 1.32)	OR: 1.03 (0.77, 1.39)	
	Doherty et al. (2017); medium	Mental Development Index (2 years)	β: −2.28 (−4.33, −0.22)	β: 1.55 (−0.39, 3.48)	0.01
	Qian et al. (2019); medium	Mental Development Index (2 years)	β: 0.31 (−1.63, 2.25)	β: 0.63 (−1.48, 2.73)	0.83
	Gascon ^a^ et al. (2015); high	Cognition (1 year)	β: 1.4 (−1.5, 4.2)	β: 0.4 (−2.4, 3.2)	
		Cognition (4 years)	β: −0.4 (−3.1, 2.4)	β: −0.4 (−3.1, 2.4)	
MEP					
	Ipapo et al. (2017); medium	Visual recognition memory (27 weeks)	β: −4.39 (−8.29, −0.49)	β:−0.69 (−3.94, 2.56)	0.55
	Doherty et al. (2017); medium	Mental Development Index (2 years)	β: −0.11 (−1.71, 1.49)	β: 0.97 (−0.46, 2.41)	0.3
	Olesen et al. (2018); medium	Vocabulary scores (20–36 months)	OR: 1.08 (0.92, 1.27)	OR: 1.24 (1.05, 1.46)	
		Language complexity scores (20–36 months)	OR: 1.10 (0.94, 1.30)	OR: 1.16 (0.97, 1.39)	
	Qian et al. (2019); medium	Mental Development Index (2 years)	β: 1.72 (−1.04, 4.49)	β: 1.03 (−1.60, 3.67)	0.73
	Gascon ^a^ et al. (2015); high	Cognition (1 year)	β: −0.8 (−3.0, 1.4)	β: −0.7 (−2.9, 1.4)	
		Cognition (4 years)	β: −0.2 (−2.2, 1.8)	β: 2.1 (−0.01, 4.2)	
MCPP					
	Ipapo et al. (2017); medium	Visual recognition memory (27 weeks)	β: −2.32 (−6.12, 1.48)	β: 2.58 (−0.69, 5.84)	0.08
	Doherty et al. (2017); medium	Mental Development Index (2 years)	β: -2.39 (-4.72, -0.05)	β: 2.03 (−0.02, 4.08)	0.007
ΣDiNPm					
	Olesen et al. (2018); medium	Vocabulary scores (20–36 months)	OR: 0.98 (0.78, 1.23)	OR: 1.18 (0.95, 1.48)	
		Language complexity scores (20–36 months)	OR: 0.88 (0.67, 1.15)	OR: 0.97, 0.76, 1.23)	
MHiNP					
	Olesen et al. (2018); medium	Vocabulary scores (20–36 months)	OR: 0.96 (0.79, 1.15)	OR: 1.16 (0.96, 1.41)	
		Language complexity scores (20–36 months)	OR: 0.93 (0.76, 1.14)	OR: 0.97 (0.80, 1.18)	
MOiNP					
	Olesen et al. (2018); medium	Vocabulary scores (20–36 months)	OR: 1.00 (0.83, 1.20)	OR: 1.19 (0.99, 1.43)	
		Language complexity scores (20–36 months)	OR: 0.90 (0.74, 1.11)	OR: 1.03 (0.85, 1.24)	
MCiOP					
	Olesen et al. (2018); medium	Vocabulary scores (20–36 months)	OR: 0.98 (0.78, 1.23)	OR: 1.17 (0.95, 1.45)	
		Language complexity scores (20–36 months)	OR: 0.88 (0.66, 1.16)	OR: 0.96 (0.76, 1.21)	

^a^ The data contained in the article Radke et al. (2020) were used. The Σ4DEHP metabolites include MEHHP, MEHP, MEOHP, MECP; * ΣDEHP_m_ molar sum of DEHP metabolites expressed as excreted DEHP ng/mL); ΣDBP: sum of molar concentrations nmol/L) of MnBP and MiBP; ΣDiNPm: molar sum of DiNP metabolites expressed as excreted DiNP ng/mL); β: beta coefficients (95% confidence intervals), OR: odds ratio (95% confidence intervals). *p***_i_**_nteraction_: *p*-value for interaction between phthalates concentrations.

**Table 4:** Sex-specific difference between phthalates exposure and child psychomotor development.

Phthalates/ Phthalate Metabolites	References/Study Confidence	Outcomes/Years	Child Psychomotor Development Effects β (95%CI)		*p*_interaction_
			Females	Males	
Σ4DEHP					
	Gascon ^a^ et al. (2015); high	Motor effects (1 year)	β: 0.5 (−3.4, 4.4)	β: 0.5 (−3.4, 4.4)	
		Motor effects (4 years)	β: 0.02 (−3.1, 3.1)	β: 0.9 (−3.0, 4.8)	
	Doherty et al. (2017); medium	Psychomotor Development Index (2 years)	β: 0.26 (−1.71, 2.24)	β: 0.22 (−1.33, 1.77)	1.0
	Balalian et al. (2019); medium	Total (BOT-2) composite score (11 years)	β: −0.21 (−1.27, 0.85)	β: −0.31 (−1.88, 1.25)	0.91
		Fine motor function (11 years)	β: −0.26 (−1.05, 0.54)	β: −0.26 (−1.28, 0.76)	1.00
		Gross motor function (11 years)	β: 0.04 (−0.47, 0.56)	β: −0.06 (−0.90, 0.79)	0.84
	Qian et al. (2019); medium	Psychomotor Development Index (2 years)	β: −1.38 (−5.30, 2.54)	β: 3.24 (0.70, 5.78)	0.05
MEHP					
	Qian et al. (2019); medium	Psychomotor Development Index (2 years)	β: 0.01 (−1.91, 1.94)	β: 2.25 (0.54, 3.96)	0.09
MEHHP					
	Qian et al. (2019); medium	Psychomotor Development Index (2 years)	β: −2.77 (−6.59, 1.06)	β: 1.91 (−0.66, 4.47)	0.05
MEOHP					
	Qian et al. (2019); medium	Psychomotor Development Index (2 years)	β: −2.89 (−6.84, 1.05)	β: 2.55 (0.05, 5.06)	0.02
MECPP					
	Qian et al. (2019); medium	Psychomotor Development Index (2 years)	β: −0.75 (−4.63, 3.12)	β: 3.49 (0.88, 6.10)	0.08
MBzP					
	Gascon ^a^ et al. (2015); high	Motor skills (1 year)	β: −2.8 (−6.0, 0.5)	β: 1.3 (−1.5, 4.2)	
		Motor skills (4 years)	β: −1.1 (−2.8, 0.7)	β: −1.4 (−4.2, 1.3)	
	Doherty et al. (2017); medium	Psychomotor Development Index (2 years)	β: −2.08 (−3.77, −0.38)	β: 0.55 (−1.21, 2.31)	0.04
	Balalian et al. (2019); medium	Total (BOT−2) composite score (11 years)	β: −1.14 (−2.13, −0.14)	β: −0.87 (−1.82, 0.07)	0.70
		Fine motor function (11 years)	β: −0.70 (−1.45, 0.04)	β: −0.79 (−1.40, −0.19)	0.85
		Gross motor function (11 years)	β: −0.44 (−0.92, 0.05)	β: −0.08 (−0.59, 0.44)	0.32
	Qian et al. (2019); medium	Psychomotor Development Index (2 years)	β: 0.13 (−1.39, 1.64)	β: 0.06 (−1.43, 1.55)	0.95
ΣDBP					
	Qian et al. (2019); medium	Psychomotor Development Index (2 years)	β: −2.60 (−5.20, 0.00)	β: −1.48 (−3.88, 0.92)	0.54
MnBP					
	Gascon ^a^ et al. (2015); high	Motor effects (1 year)	β: 0.6 (−2.6, 3.7)	β: 1.8 (−1.7, 5.3)	
		Motor effects (4 years)	β: 0.2 (−2.3, 2.6)	β: 0.2 (−3.3, 3.6)	
	Doherty et al. (2017); medium	Psychomotor Development Index (2 years)	β: −2.29 (−4.63, 0.05)	β: 1.92 (0.31, 3.54)	0.005
	Balalian et al. (2019); medium	Total BOT-2) composite score (11 years)	β: −2.09 (−3.43, −0.75)	β: −0.63 (−1.96, 0.69)	0.13
		Fine motor function (11 years)	β: −1.43 (−2.44, −0.42)	β: −0.78 (−1.63, 0.07)	0.33
		Gross motor function (11 years)	β: −0.66 (−1.33, 0.002)	β: 0.15 (−0.56, 0.86)	0.10
	Qian et al. (2019); medium	Psychomotor Development Index (2 years)	β: −2.30 (−4.67, 0.06)	β: −1.75 (−3.80, 0.31)	0.73
MiBP					
	Doherty et al. (2017); medium	Psychomotor Development Index (2 years)	β: −0.65 (−2.87, 1.58)	β: 0.68 (−1.24, 2.6)	0.4
	Balalian et al. (2019); medium	Total BOT-2) composite score (11 years)	β: −1.36 (−2.51, −0.21)	β: −0.58 (−1.93, 0.77)	0.38
		Fine motor function (11 years)	β: −0.80 (−1.66, 0.07)	β: −0.54 (−1.42, 0.33)	0.68
		Gross motor function (11 years)	β: −0.56 (−1.12,−0.01)	β: −0.03 (−0.76, 0.69)	0.25
	Qian et al. (2019); medium	Psychomotor Development Index (2 years)	β: −0.44 (−2.23, 1.35)	β: 0.40 (−1.25,2,05)	0.50
	Gascon ^a^ et al. (2015); high	Motor effects (1 year)	β: −0.9 (−4.4, 2.7)	β: −0.8 (−3.9, 2.4)	
		Motor effects (4 years)	β: −1.8 (−4.6, 1.0)	β: −2.0 (−5.1, 1.1)	
MEP					
	Doherty et al. (2017); medium	Psychomotor Development Index (2 years)	β: 0.63 (−1.08, 2.34)	β: 0.19 (−1.25, 1.63)	0.7
	Balalian et al. (2019); medium	Total BOT-2) composite score (11 years)	β: −1.23 (−2.36, −0.11)	β: 0.50 (−0.52, 1.52)	0.02
		Fine motor function (11 years)	β: −0.63 (−1.48, 0.22)	β: 0.25 (−0.42, 0.91)	0.11
		Gross motor function (11 years)	β: −0.60 (−1.14, −0.06)	β: 0.25 (−0.29, 0.80)	0.03
	Qian et al. (2019); medium	Psychomotor Development Index (2 years)	β: 0.08 (−2.48, 2.65)	β: 0.36 (−1.71, 2.43)	0.87
	Gascon ^a^ et al. (2015); high	Motor skills (1 year)	β: −0.2 (−3.6, 1.3)	β: −1.2 (−3.7, 1.2)	
		Motor skills (4 years)	β: 2.0 (−0.4, 4.4)	β: 0.5 (−1.8, 2.7)	
MCPP					
	Doherty et al. (2017); medium	Psychomotor Development Index (2 years)	β: −2.93 (−5.35, −0.51)	β: 1.61 (−0.42, 3.64)	0.006
MCiOP					
	Balalian et al. (2019) medium	Total BOT-2) composite score (11 years)	β: −4.34 (−9.06, 0.37)	β: −0.92 (−3.17, 1.32)	0.18
		Fine motor function (11 years)	β: −3.27 (−7.00, 0.45)	β: −0.84 (−3.17, 1.32)	0.21
		Gross motor function (11 years)	β: −1.07 (−2.98, 0.81)	β: −0.08 (−1.32, 1.16)	0.36
non-DEHP					
	Daniel et al. (2020); medium	Gross motor function (11 years)	β: −0.98 (−1.98, 0.03)	NA	
		Fine motor function (11 years)	β: −0.85 (−1.49, −0.20)	NA	

^a^ The data contained in the article Radke et al. (2020) were used; the Σ4DEHP metabolites include MEHHP, MEHP, MEOHP, MECP; ΣDBP: sum of molar concentrations nmol/L of MnBP and MiBP; non-DEHP: MBP, MEP, MiBP, MBzP) NA: no associations; β: beta coefficients (95% confidence Intervals); *p*_interaction_: *p*-value for interaction between phthalates concentrations.

**Table 5:** Sex-specific differences between phthalates exposure and child behavioural and emotional problems.

Phthalates/Phthalate Metabolites	References/Study Confidence	Outcomes/Years	Child Behavioural and Emotional Problems β (95%CI), OR (95%CI), MR (95%CI)		*p*_interaction_
			Females	Males	
ΣDEHP					
	Gascon ^a^ et al. (2015); hight	Behavioural problems (7 years)	NA	NA	
	Li ^#^ et al. (2020); high	Internalizing problems (1, 2, 3, 4, 5 and 8 years)	β: 0.5 (−1.0, 1.9)	β: −0.6 (−2.1, 0.9)	0.77
		Externalizing problems (1, 2, 3, 4, 5 and 8 years)	β: 0.1 (−1.2, 1.4)	β: −0.5 (−1.8, 0.9)	0.97
		Behavioural Symptoms Index (1, 2, 3, 4, 5 and 8 years)	β: 0.3 (−1.0, 1.5)	β: −0.1 (−1.5, 1.4)	0.99
	Percy * et al. (2016); high	Typical play behaviors (8 years)	OR: 1.15 (0.81, 1.63)	OR: 1.11 (0.75, 1.63)	
	Oulhote ^^^ et al. (2020); medium	Autistic traits (3–4 years)	β: −0.3 (−1.0, 0.4)	β: 0.4 (−0.3, 1.1)	0.17
ΣMEHP					
	Huang ^□^ et al. (2019); medium	Internalizing problems (8–9, 11–12, 14–15 years)	β: 0.02 (−0.03, 0.06)	β: 0.01 (−0.02, 0.04)	0.67
		Externalizing problems (8–9, 11–12, 14–15 years)	β: 0.02 (−0.02, 0.06)	β: 0.03 (−0.007, 0.06)	0.90
MEHP					
	Lien et al. (2015); medium	Internalizing problems (8–9 years)	β: 2.41 (−3.26, 8.08)	β: 0.55 (−4.30, 5.39)	
		Externalizing problems (8–9 years)	β: 5.88 (0.66, 11.09)	β: 1.81 (−4.75, 8.38)	
	Percy et al. (2016); high	Typical play behaviors (8 years)	OR: 1.09 (0.81, 1.47)	OR: 0.95 (0.66, 1.37)	
	Huang et al. (2019); medium	Internalizing problems (8–9, 11–12, 14–15 years)	β: 0.03 (−0.01, 0.08)	β: 0.02 (−0.02, 0.06)	0.74
		Externalizing problems (8–9, 11–12, 14–15 years)	β: 0.04 (−0.0005, 0.07)	β: 0.04 (−0.0005, 0.08)	0.77
	Daniel et al. (2020b); medium	Anxious–shy (7 years)	MR: 0.94 (0,84, 1.06)	MR: 1.07 (0.93, 1.23)	
		Perfectionism (7 years)	MR: 0.97 (0.87, 1.09)	MR: 1.03 (0.92, 1.16)	
		Social problems (7 years)	MR: 0.91 (0.74, 1.11)	MR: 1.35 (1.07, 1.7)	
		Psychosomatic problems (7 years)	MR: 1.02 (0.87, 1.18)	MR: 1.09 (0.92, 1.29)	
		Emotional lability (7 years)	MR: 0.95 (0.81, 1.11)	MR: 0.99 (0.84, 1.17)	
		Cognitive problems (7 years)	MR: 0.88 (0.77, 1.01)	MR: 1.03 (0.9,1.18)	
		Oppositional problems (7 years)	MR: 0.98 (0.84, 1.13)	MR: 1.04 (0.92, 1.17)	
		Hyperactivity (7 years)	MR: 0.84 (0.73, 0.95)	MR: 0.95 (0.85, 1.07)	
		Impulsiveness–restless (7 years)	MR: 0.89 (0.78, 1.02)	MR: 0.98 (0.86, 1.11)	
		Global index (7 years)	MR: 0.9 (0.8, 1.03)	MR: 0.97 (0.86, 1.1)	
		ADHD index (7 years)	MR: 0.87 (0.75, 1)	MR: 1 (0.88, 1.13)	
	England-Mason et al. (2020); medium	Internalizing problems (3–4 years)	β: −0.11 (−0.28, 0.07)	β: −0.01 (−0.18, 0.16)	0.36
		Externalizing problems (3–4 years)	β: −0.08 (−0.24, 0.08)	β: 0.10 (−0.07,0.27)	0.12
		Behavioural Symptoms Index (3–4 years)	β: −0.02 (−0.19, 0.15)	β: 0.09 (−0.07, 0.25)	0.28
MEHHP					
	Lien et al. (2015); medium	Internalizing problems (8–9 years)	β: 0.82 (−2.01, 3.65)	β: 0.03 (−2.51, 2.57)	
		Externalizing problems (8–9 years)	β: 1.71 (−0.96, 4.38)	β: 0.95 (−2.49, 4.39)	
	Percy et al. (2016); high	Typical play behaviors (8 years)	OR: 1.10 (0.79, 1.52)	OR: 1.10 (0.76, 1.60)	
	Daniel et al. (2020b); medium	Anxious–shy (7 years)	MR: 0.97 (0.85, 1.12)	MR: 1.02 (0.86, 1.22)	
		Perfectionism (7 years)	MR: 0.98 (0.86, 1.11)	MR: 1.01 (0.87, 1.16)	
		Social problems (7 years)	MR: 0.82 (0.64, 1.05)	MR: 1.18 (0.89, 1.57)	
		Psychosomatic problems (7 years)	MR: 0.99 (0.83, 1.18)	MR: 1.03 (0.83, 1.26)	
		Emotional lability (7 years)	MR: 0.96 (0.79, 1.16)	MR: 0.88 (0.71, 1.08)	
		Cognitive problems (7 years)	MR: 0.93 (0.79, 1.09)	MR: 0.93 (0.79, 1.1)	
		Oppositional problems (7 years)	MR: 1 (0.84, 1.18)	MR: 0.98 (0.84, 1.13)	
		Hyperactivity (7 years)	MR: 0.85 (0.72, 0.99)	MR: 0.94 (0.81, 1.08)	
		Impulsiveness–restless (7 years)	MR: 0.9 (0.77, 1.06)	MR: 0.93 (0.8, 1.08)	
		Global index (7 years)	MR: 0.92 (0.79, 1.06)	MR: 0.91 (0.79, 1.06)	
		ADHD index (7 years)	MR: 0.92 (0.77, 1.09)	MR: 0.93 (0.79, 1.09)	
	England-Mason et al. (2020); medium	Internalizing problems (3–4 years)	β:−0.06 (−0.26, 0.14)	β: 0.02 (−0.18, 0.22)	0.46
		Externalizing problems (3–4 years)	β: −0.04 (−0.22, 0.14)	β: −0.01 (−0.20, 0.19)	0.51
		Behavioural Symptoms Index (3–4 years)	β: −0.02 (−0.21, 0.17)	β: 0.05 (−0.14,0.24)	0.33
MEOHP					
	Lien et al. (2015); medium	Internalizing problems (8–9 years)	β: 0.72 (−2.92, 4.36)	β: 1.33 (−1.45, 4.11)	
		Externalizing problems (8–9 years)	β: 3.68 (0.35, 7.02)	β: 3.73 (0.07, 7.39)	
	Percy et al. (2016); high	Typical play behaviors (8 years)	OR: 1.12 (0.80, 1.56)	OR: 1.13 (0.77, 1.65)	
	Daniel et al. (2020b); medium	Anxious–shy (7 years)	MR: 0.95 (0.82, 1.09)	MR: 1.06 (0.89, 1.26)	
		Perfectionism (7 years)	MR: 0.99 (0.86, 1.13)	MR: 1.02 (0.88, 1.19)	
		Social problems (7 years)	MR: 0.8 (0.62, 1.03)	MR: 1.18 (0.89, 1.58)	
		Psychosomatic problems (7 years)	MR: 0.98 (0.81, 1.17)	MR: 1.06 (0.86, 1.32)	
		Emotional lability (7 years)	MR: 0.94 (0.77, 1.15)	MR: 0.88 (0.7, 1.09)	
		Cognitive problems (7 years)	MR: 0.92 (0.77, 1.08)	MR: 0.94 (0.79, 1.12)	
		Oppositional problems (7 years)	MR: 0.98 (0.83, 1.16)	MR: 0.98 (0.84, 1.14)	
		Hyperactivity (7 years)	MR: 0.83 (0.71, 0.98)	MR: 0.94 (0.81, 1.09)	
		Impulsiveness–restless (7 years)	MR: 0.86 (0.73, 1.02)	MR: 0.92 (0.78, 1.08)	
		Global index (7 years)	MR: 0.89 (0.76, 1.03)	MR: 0.91 (0.78, 1.06)	
		ADHD index (7 years)	MR: 0.89 (0.74, 1.06)	MR: 0.93 (0.78, 1.1)	
	England-Mason et al. (2020); medium	Internalizing problems (3–4 years)	β: −0.08 (−0.28, 0.12)	β: 0.07 (−0.14, 0.27)	0.28
		Externalizing problems (3–4 years)	β:−0.03 (−0.21, 0.15)	β: −0.06 (−0.26, 0.14)	0.76
		Behavioural Symptoms Index (3–4 years)	β: −0.03 (−0.23, 0.16)	β: 0.02 (−0.17, 0.22)	0.40
MECPP					
	Percy et al. (2016); high	Typical play behaviors (8 years)	OR: 1.20 (0.82,1.74)	OR: 1.11 (0.72, 1.63)	
	Daniel et al. (2020b); medium	Anxious–shy (7 years)	MR: 0.95 (0.81, 1.11)	MR: 1.09 (0.91, 1.32)	
		Perfectionism (7 years)	MR: 1 (0.86, 1.15)	MR: 1.04 (0.89, 1.22)	
		Social problems (7 years)	MR: 0.78 (0.58, 1.04)	MR: 1.2 (0.88, 1.63)	
		Psychosomatic problems (7 years)	MR: 0.97 (0.79, 1.19)	MR: 1.04 (0.82, 1.31)	
		Emotional lability (7 years)	MR: 0.89 (0.71, 1.12)	MR: 0.9 (0.71, 1.13)	
		Cognitive problems (7 years)	MR: 0.89 (0.74, 1.07)	MR: 0.93 (0.77, 1.12)	
		Oppositional problems (7 years)	MR: 0.97 (0.79, 1.18)	MR:1.01 (0.85, 1.19)	
		Hyperactivity (7 years)	MR: 0.84 (0.7, 1.01)	MR: 0.91 (0.78, 1.07)	
		Impulsiveness–restless (7 years)	MR: 0.92 (0.77, 1.1)	MR: 0.91 (0.77, 1.08)	
		Global Index (7 years)	MR: 0.91 (0.76, 1.08)	MR: 0.91 (0.77, 1.07)	
		ADHD Index (7 years)	MR: 0.92 (0.76, 1.12)	MR: 0.92 (0.77, 1.1)	
	England-Mason et al. (2020); medium	Internalizing problems (3–4 years)	β: −0.06 (−0.27, 0.14)	β: 0.03 (−0.17, 0.23)	0.48
		Externalizing problems (3–4 years)	β: −0.06 (−0.25, 0.13)	β: −0.03 (−0.23, 0.16)	0.62
		Behavioural Symptoms Index (3–4 years)	β: −0.06 (−0.26, 0.14)	β: 0.01 (−0.19, 0.20)	0.42
MMP					
	Lien et al. (2015); medium	Internalizing problems (8–9 years)	β: −0.35 (−5.40, 4.71)	β: −2.79 (−6.83, 1.25)	
		Externalizing problems (8–9 years)	β: 3.54 (−1.19, 8.27)	β: −5.22 (−10.62, 0.18)	
	Huang et al. (2019); medium	Internalizing problems (8–9, 11–12, 14–15 years)	β: −0.02 (−0.05, 0.02)	β: −0.02 (−0.05, 0.01)	0.89
		Externalizing problems (8–9, 11–12, 14–15 years)	β: −0.02 (−0.05, 0.01)	β: −0.02 (−0.06,0.01)	0.76
	England-Mason et al. (2020); medium	Internalizing problems (3–4 years)	β: −0.04 (−0.22, 0.13)	β: 0.17 (−0.01, 0.34)	0.10
		Externalizing problems (3–4 years)	β: −0.02 (−0.19, 0.14)	β: 0.11 (−0.06, 0.29)	0.21
		Behavioural Symptoms Index (3–4 years)	β: −0.11 (−0.29, 0.06)	β: 0.04 (−0.13, 0.21)	0.16
MEP					
	Gascon ^a^ et al. (2015); hight	Behavioural problems (7 years)	NA	NA	
	Huang et al. (2019); medium	Internalizing problems (8–9, 11–12, 14–15 years)	β: −0.002 (−0.04, 0.04)	β: −0.003 (−0.05, 0.04)	0.79
		Externalizing problems (8–9, 11–12, 14–15 years)	β: −0.01 (−0.05, 0.03)	β: −0.005 (−0.05, 0.04)	0.63
	Li et al. (2020); high	Internalizing problems (1, 2, 3, 4, 5 and 8 years)	β: 0.2 (−1.2, 1.5)	β: −0.1 (−1.4, 1.2)	0.41
		Externalizing problems (1, 2, 3, 4, 5 and 8 years)	β: 0.0 (−1.3, 1.4)	β: −1.2 (−2.6, 0.1)	0.15
		Behavioural Symptoms Index (1, 2, 3, 4, 5 and 8 years)	β: −0.3 (−1.5, 1.0)	β: −1.1 (−2.5, 0.2)	0.36
	Lien et al. (2015); medium	Internalizing problems (8–9 years)	β: −2.95 (−8.60, 2.70)	β: −0.16 (−5.16, 4.84)	
		Externalizing problems (8–9 years)	β: 0.08 (−5.36, 5.53)	β: −1.61 (−8.38, 5.16)	
	Percy et al. (2016); high	Typical play behaviors (8 years)	OR: 0.70 (0.51, 0.97)	OR: 0.72 (0.51, 1.02)	
	Daniel et al. (2020b); medium	Anxious–shy (7 years)	MR: 0.88 (0.76, 1.03)	MR: 1.04 (0.89, 1.21)	
		Perfectionism (7 years)	MR: 0.95 (0.82, 1.09)	MR: 1.1 (0.97, 1.25)	
		Social problems (7 years)	MR: 1.18 (0.92, 1.5)	MR: 1.13 (0.86, 1.47)	
		Psychosomatic problems (7 years)	MR: 0.97 (0.8, 1.17)	MR: 0.93 (0.77, 1.13)	
		Emotional lability (7 years)	MR: 0.92 (0.74, 1.15)	MR: 1.08 (09, 1.3)	
		Cognitive problems (7 years)	MR: 1.03 (0.87, 1.22)	MR: 0.95 (0.82, 1.1)	
		Oppositional problems (7 years)	MR: 1.04 (0.87, 1.26)	MR: 1 (0.84, 1.14)	
		Hyperactivity (7 years)	MR: 0.98 (0.82, 1.16)	MR: 1.07 (0.94, 1.21)	
		Impulsiveness–restless (7 years)	MR: 0.94 (0.79, 1.13)	MR: 1 (0.87, 1.14)	
		Global Index (7 years)	MR: 0.94 (0.79, 1.11)	MR: 1.02 (0.89, 1.16)	
		ADHD Index (7 years)	MR: 0.98 (0.81, 1.19)	MR: 0.96 (0.83, 1.11)	
	Oulhote et al. (2020); medium	Autistic traits (3–4 years)	β: −0.4 (−0.7, 0.0)	β: 0.2 (−0.2, 0.5)	0.04
	England-Mason et al. (2020); medium	Internalizing problems (3–4 years)	β: −0.05 (−0.23, 0.12)	β: 0.08 (−0.08, 0.24)	0.28
		Externalizing problems (3–4 years)	β: 0.01 (−0.16, 0.17)	β: 0.08 (−0.08, 0.23)	0.46
		Behavioural Symptoms Index (3–4 years)	β: −0.01 (−0.18, 0.17)	β: 0.08 (−0.07, 0.23)	0.35
MnBP					
	Li et al. (2020); high	Internalizing problems (1, 2, 3, 4, 5 and 8 years)	β: −0.2 (−1.5, 1.0)	β: −0.7 (−0.2, 0.5)	0.61
		Externalizing problems (1, 2, 3, 4, 5 and 8 years)	β: 0.5 (−0.9, 1.8)	β: 0.4 (−0.9, 1.7)	0.95
		Behavioural Symptoms Index (1, 2, 3, 4, 5 and 8 years)	β: −0.2 (−1.4, 1.0)	β: −0.4 (−1.5, 0.8)	0.89
	Percy et al. (2016); high	Typical play behaviors (8 years)	OR: 1.01 (0.63, 1.62)	OR: 1.44 (0.82, 2.53)	
	Daniel et al. (2020b); medium	Anxious–shy (7 years)	MR: 1.02 (0.85, 1.22)	MR: 1.15 (0.96, 1,38)	
		Perfectionism (7 years)	MR: 1.1 (0.93, 1.3)	MR: 1.04 (0.89, 1.21)	
		Social problems (7 years)	MR: 1.12 (0.82, 1.54)	MR: 1.34 (0.99, 1.82)	
		Psychosomatic problem (7 years)	MR: 1.28 (1.02, 1.59)	MR: 1.11 (0.89, 1.38)	
		Emotional lability (7 years)	MR: 1.06 (0.83, 1.36)	MR: 0.87 (0.7, 1.08)	
		Cognitive problems (7 years)	MR: 1.17 (0.95, 1.43)	MR: 0.97 (0.82,1.16)	
		Oppositional problems (7 years)	MR: 1.06 (0.85,1.33)	MR: 0.98 (0.83, 1.14)	
		Hyperactivity (7 years)	MR: 1.08 (0.92, 1.26)	MR: 0.99 (0.85, 1.16)	
		Impulsiveness–restless (7 years)	MR: 1.03 (0.84, 1.27)	MR: 0.97 (0.82, 1.14)	
		Global Index (7 years)	MR: 1.04 (0.86, 1.27)	MR: 0.94 (0.8, 1.1)	
		ADHD Index (7 years)	MR: 1.12 (0.9, 1.39)	MR: 0.94 (0.79, 1.12)	
MBP					
	Lien et al. (2015); medium	Internalizing problems (8–9 years)	β: 3.03 (−1.76, 7.81)	β: 0.06 (−5.12, 5.25)	
		Externalizing problems (8–9 years)	β: 7.63 (3.46, 11.80)	β: −2.51 (−9.51, 4.50)	
	Huang et al. (2019); medium	Internalizing problems (8–9, 11–12, 14–15 years)	β: 0.02 (−0.02, 0.05)	β: 0.01 (−0.03, 0.05)	0.83
		Externalizing problems (8–9, 11–12, 14–15 years)	β: 0.03 (−0.006, 0.06)	β: 0.01 (−0.04, 0.05)	0.59
	England-Mason et al. (2020); medium	Internalizing problems (3–4 years)	β: 0.07 (−0.14, 0.27)	β: 0.15 (−0.05, 0.34)	0.50
		Externalizing problems (3–4 years)	β: 0.10 (−0.09, 0.29)	β: 0.13 (−0.06, 0.33)	0.20
		Behavioural Symptoms Index (3–4 years)	β: 0.04 (−0.16, 0.24)	β: 0.19 (0.01, 0.37)	0.20
	Gascon ^a^ et al. (2015); hight	Behavioural problems (7 years)	NA	NA	
	Oulhote et al. (2020); medium	Autistic traits (3–4 years)	β: 0.1 (−0.6, 0.7)	β: 1.0 (0.4, 1.6)	0.03
MiBP					
	Gascon ^a^ et al. (2015); hight	Behavioural problems (7 years)	NA	NA	
	Li et al. (2020); high	Internalizing problems (1, 2, 3, 4, 5 and 8 years)	β: 0.2 (−1.2, 1.6)	β:0.0 (−1.4, 1.4)	0.40
		Externalizing problems (1, 2, 3, 4, 5 and 8 years)	β: 0.6 (−0.7, 1.9)	β: 0.8 (−0.5, 2.2)	0.83
		Behavioural Symptoms Index (1, 2, 3, 4, 5 and 8 years)	β: 0.0 (−1.3, 1.2)	β: 0.6 (−0.7, 1.8)	0.73
	Percy et al. (2016); high	Typical play behaviors (8 years)	OR: 0.68 (0.41,1.14)	OR: 1.69 (1.00, 2.86)	
	Daniel et al. (2020b); medium	Anxious–shy (7 years)	MR: 0.92 (0.77, 1.08)	MR: 1.22 (1.02, 1.47)	
		Perfectionism (7 years)	MR: 1.03 (0.87, 1.2)	MR: 1.04 (0.89, 1.21)	
		Social problems (7 years)	MR: 1.01 (0.74, 1.38)	MR: 1.15 (0.83, 1.59)	
		Psychosomatic problems (7 years)	MR: 1.1 (0.88, 1.37)	MR: 1.28 (1.02, 1.6)	
		Emotional lability (7 years)	MR: 1.13 (0.88, 1.45)	MR: 1 (0.81, 1.25)	
		Cognitive problems (7 years)	MR: 1.07 (0.87, 1.31)	MR: 1.09 (0.91, 1.3)	
		Oppositional problems (7 years)	MR: 1.09 (0.88, 1.37)	MR: 1.07 (0.9, 1.26)	
		Hyperactivity (7 years)	MR: 1 (0.82, 1.23)	MR: 1.01 (0.86, 1.17)	
		Impulsiveness–restless (7 years)	MR: 0.95 (0.78, 1.16)	MR: 1.03 (0.87, 1.21)	
		Global Index (7 years)	MR: 1 (0.82, 1.21)	MR: 1.02 (0.87, 1.19)	
		ADHD Index (7 years)	MR: 1.04 (0.83, 1.29)	MR: 1.05 (0.89, 1.25)	
	England-Mason et al. (2020); medium	Internalizing problems (3–4 years)	β: 0.20 (−0.05, 0.44)	β: 0.21 (0.01, 0.41)	0.72
		Externalizing problems (3–4 years)	β: 0.22 (−0.01, 0.45)	β: 0.12 (−0.09, 0.32)	0.95
		Behavioural Symptoms Index (3–4 years)	β: 0.20 (−0.05, 0.44)	β: 0.22 (0.03, 0.42)	0.48
MBzP					
	Gascon ^a^ et al. (2015); hight	Behavioural problems (7 years)	NA	NA	
	Huang et al. (2019); medium	Internalizing problems (8–9, 11–12, 14–15 years)	β: −0.03 (−0.08, 0.03)	β: 0.005 (−0.04, 0.05)	0.40
		Externalizing problems (8–9, 11–12, 14–15 years)	β: −0.02 (−0.06, 0.03)	β: 0.003 (−0.05, 0.05)	0.71
	Li et al. (2020); high	Internalizing problems (1, 2, 3, 4, 5 and 8 years)	β: 0.5 (−1.0, 2.0)	β: 0.0 (−1.3, 1.2)	0.36
		Externalizing problems (1, 2, 3, 4, 5 and 8 years)	β: 0.9 (−0.8, 2.6)	β: 0.8 (−0.5, 2.2)	0.60
		Behavioural Symptoms Index (1, 2, 3, 4, 5 and 8 years)	β: 0.9 (−0.9, 2.6)	β: 0.4 (−0.8, 1.7)	0.58
	Lien et al. (2015); medium	Internalizing problems (8–9 years)	β: −2.78 (−10.32, 4.75)	β: −2.66 (−8.23, 2.92)	
		Externalizing problems (8–9 years)	β: 1.99 (−5.22, 9.19)	β: −4.24 (−11.79,3.31)	
	Percy et al. (2016); high	Typical play behaviors (8 years)	OR: 1.26 (0.88, 1.81)	OR: 1.39 (0.91, 2.11)	
	Daniel et al. (2020b); medium	Anxious–shy (7 years)	MR: 1.04 (0.91, 1.19)	MR: 1.20 (1.05, 1.36)	
		Perfectionism (7 years)	MR: 1.15 (1.01, 1.30)	MR: 1.04 (0.92, 1.17)	
		Social problems (7 years)	MR: 1.01 (0.79, 1.28)	MR: 1.02 (0.8, 1.31)	
		Psychosomatic problems (7 years)	MR: 1.04 (0.87, 1.24)	MR: 1.15 (0.97, 1.35)	
		Emotional lability (7 years)	MR: 1.06 (0.88, 1.27)	MR: 1.03 (0.88,1.22)	
		Cognitive problems (7 years)	MR: 1.17 (1, 1.37)	MR: 1.02 (0.89, 1.17)	
		Oppositional problems (7 years)	MR: 1.09 (0.92, 1.3)	MR: 1.07 (0.95, 1.2)	
		Hyperactivity (7 years)	MR: 1.08 (0.92, 1.26)	MR: 0.99 (0.88, 1.11)	
		Impulsiveness–restless (7 years)	MR: 1.07 (0.92, 1.25)	MR: 0.96 (0.85, 1.09)	
		Global Index (7 years)	MR: 1.07 (0.92, 1.24)	MR: 0.98 (0.87, 1.11)	
		ADHD Index (7 years)	MR: 1.1 (0.94, 1.3)	MR: 1 (0.88, 1.14)	
	England-Mason et al. (2020); medium	Internalizing problems (3–4 years)	β: 0.09 (−0.09, 0.28)	β: 0.24 (0.06, 0.42)	0.28
		Externalizing problems (3–4 years)	β: 0.08 (−0.09, 0.25)	β: 0.26 (0.08, 0.44)	0.16
		Behavioural Symptoms Index (3–4 years)	β: 0.10 (−0.08, 0.28)	β: 0.26 (0.09, 0.43)	0.21
	Oulhote et al. (2020); medium	Autistic traits (3–4 years)	β: 0.1 (−0.5, 0.6)	β: 0.3 (−0.3, 0.8)	0.60
MCPP					
	Li et al. (2020); high	Internalizing problems (1, 2, 3, 4, 5 and 8 years)	β: 1.2 (−0.2, 2.7)	β: 0.7 (−0.8, 2.1)	0.96
		Externalizing problems (1, 2, 3, 4, 5 and 8 years)	β: 1.5 (0.1, 3.0)	β: 0.6 (−0.9, 2.0)	0.78
		Behavioural Symptoms Index (1, 2, 3, 4, 5 and 8 years)	β: 1.1 (−0.3, 2.4)	β: 1.2 (−0.3, 2.7)	0.58
	Percy et al. (2016); high	Typical play behaviors (8 years)	OR: 0.67 (0.35, 1.28)	OR: 1.26 (0.72, 2.20)	
	Oulhote et al. (2020); medium	Autistic traits (3–4 years)	β: 0.3 (−0.2, 0.9)	β: 0.6 (0.1, 1.0)	0.50

^a^ The data contained in the article Radke et al., (2020) were used; β: beta coefficients (95% Confidence Intervals), OR: odds ratio (95% confidence intervals); mean ratio (95% confidence intervals); * ΣDEHP expressed as MEHP, ng/mL (=molar sum of (MECPP, MEOHP, MEOHP, MEHP)); ^ ΣDEHP sum of MEHP, MEHHP, MEOHP; ^#^ Σ4DEHP sum of MEHHP, MEOHP, MECPP, MEHP; ^□^ ΣMEHP = MEHP + MEHHP + MEOHP; *p***_i_**_nteraction_: *p*-value for interaction between phthalates concentrations; NA^:^ no associations.

## Supplementary Materials

The following are available online at https://www.mdpi.com/article/10.3390/ijerph182413013/s1, Table S1: selected phthalates and their respective metabolites (primary and secondary); Table S2: confounders adjusted for the model’s study; Table S3: evaluation of the studies (based on methodology developed by Radke et al. 2020).
