# Supplementary material for: Review of the Existing Evidence for Sex-Specific Relationships between Prenatal Phthalate Exposure and Children’s Neurodevelopment
Source: Int J Environ Res Public Health. 2021 Dec 9;18(24):13013. doi: 10.3390/ijerph182413013 (PMC8700807; doi:10.3390/ijerph182413013)
Supplement: Supplementary file 1 [file ijerph-18-13013-s001.zip › ijerph-1442031-supplementary.pdf]

Supplementary Materials

Review of the Existing Evidence for Sex-Specific Relationships between Prenatal Phthalate Exposure and Children's Neurodevelopment

Agnieszka Jankowska, Linda Nazareth, Dorota Kaleta and Kinga Polańska

Table S1. Selected phthalates and their respective metabolites (primary and secondary)

Phthalate	Primary metabolite	Secondary metabolite
**High-molecular weight phthalates**		
Di(2-ethylhexyl) phthalate (DEHP)	Mono-2-ethylhexyl phthalate (MEHP)	5OH-Mono(2-ethylhexyl) phthalate (5OH-MEHP / MEHHP)<br>5oxo-Mono(2-ethylhexyl) phthalate (5oxo-MEHP / MEOHP)<br>5carboxy-Mono(2-ethylhexyl) phthalate (5cx-MEPP / MECPP)
Di-n-octyl phthalate (DnOP)	Mono-n-octyl phthalate (MnOP)<br>3carboxy-Mono-propyl phthalate (MCPP*)	
Di-iso-nonyl phthalate (DiNP)	Mono-iso-nonyl phthalate (MiNP)	7OH-Mono-methyloctyl phthalate (OH-MiNP / MHiNP)<br>7oxo-Mono-methyloctyl phthalate (oxo-MiNP / MOiNP)<br>7carboxy-Mono-methylheptyl phthalate (cx-MiNP / MCiOP)
**Low-molecular weight phthalates**		
Di-methyl phthalate (DMP)	Mono-methyl phthalate (MMP)	
Di-ethyl phthalate (DEP)	Mono-ethyl phthalate (MEP)	
Di-iso-butyl phthalate (DiBP)	Mono-iso-butyl phthalate (MiBP)	3OH-Mono-methylpropyl phthalate (OH-MiBP)
Di-n-butyl phthalate (DnBP)	Mono-n-butyl phthalate (MnBP)	3OH-Mono-n-butyl phthalate (OH-MnBP)<br>3carboxy-Mono-propyl phthalate (3cx-MiBP / MCPP*)
Butyl-benzyl phthalate (BBzP)	Mono-benzyl phthalate (MBzP)	

*MCPP is major metabolite of DnOP, secondary metabolite of DnBP and a non-specific metabolite of various high molecular weight phthalates

Table S2. Confounders adjusted for model’s study

References	Confounding factors
Gascon et al., (2015)	BSID - sex, maternal education, maternal smoking during pregnancy, birth season, breastfeeding, country origin of the mother, number of siblings, maternal age.<br>MCSA - age of the child at the time of outcome assessment, sex, maternal education, maternal smoking during pregnancy, birth season, breastfeeding, country origin of the mother, number of siblings, maternal age.
Lien et al., (2015)	Children’s sex, IQ and family income.
Huang et al., (2019)	Children's sex, IQ, family income, and study visit.
Percy et al., (2016)	Multivariable models for GIQ included child race, mother’s education and relational frustration score as covariates.<br>Multivariable models for PPPSI included child race and mother’s education as covariates.
Braun et al., (2017)	Maternal race, maternal age at delivery, marital status, maternal education, parity, household income, serum cotinine concentrations during pregnancy, binge drinking during pregnancy, depressive symptoms during pregnancy, prenatal vitamin use, maternal IQ and child age at testing.
Li et al., (2020)	Maternal age at delivery, pre-pregnancy BMI, maternal gestational smoking, mother’s Beck's Depression Inventory score, gestational alcohol consumption, maternal education, marital status, and child’s sex, race, and age at outcome assessment.
Ipapo et al., (2017)	Maternal specific gravity, infant sex, maternal ethnicity, maternal age at birth, maternal education, postnatal age.
Balalian et al., (2019)	Prenatal specific gravity, maternal ethnicity, prenatal maternal demoralization, prenatal maternal alcohol consumption, quality of the home environment, child BMI z-score at age 11, and child's age in months at BOT-2 administration.
Daniel et al., (2020)	Child age and BMI z-score at the time of BOT-2 performance, maternal race, prenatal alcohol consumption, maternal demoralization score and HOME score. To account for urinary dilution, the models were adjusted for urine's specific gravity as a covariate
Daniel et al., (2020b)	Maternal race/ethnicity, maternal demoralization at child age 7 years, child age at time of CPRS or CBCL assessment, prenatal specific gravity, and CAARS inattention/memory (for CPRS

	outcomes only).
Doherty et al., (2017)	Urine creatinine concentration, maternal ethnicity, pre-pregnancy BMI, maternal age in years, maternal education, supportive qualities of child's home environment, child's gender, child's age at testing in months, maternal marital status, duration of breastfeeding in months.
Kim et al., (2018)	Maternal age, birth delivery mode, gestational age, primparous, monthly household income, child's sex, and BDI (Beck Depression Inventory) of mothers.
Olesen et al., (2018)	Maternal age, maternal education.
Daniel et al., (2020b)	Maternal race/ethnicity, maternal demoralization at child age 7 years, child age at time of CPRS or CBCL assessment, prenatal specific gravity, and CAARS inattention/memory (for CPRS outcomes only).
Hyland et al., (2019)	Maternal age at enrollment, education, country of birth, years in the United States, depression at time of assessment, child sex, child age at time of assessment, language of assessment, HOME score, and household income at time of assessment
Qian et al., (2019)	Maternal age, maternal education, pre-pregnancy BMI, gestational weight gain, passive smoking and folic acid supplement during pregnancy, parity, and breast-feeding status
England - Mason et al., (2020)	Urinary creatinine, family income, child sex, and Full-Scale Intelligence Quotient (FSIQ)
Oulhote et al., (2020)	Child sex, folic acid supplementation, study site (city), year of enrollment, household income, maternal education, marital status, race/ethnicity, maternal age, and parity

BSID: Bayley Scales of Infant and Toddler Development; MSCA: McCarthy Scales of Children's Abilities; GIQ: Gender Identity Questionnaire; PPPSI: Playmate and play style preferences structured Interview; BOT-2 Bruininks - Oseretsky Test of Motor Proficiency - 2nd edition; CPRS: The Conners' Parent Rating Scale-Revised: Long Form; CBCL: Child Behavior Checklist.

Table S3. Evaluation of the studies (based on methodology developed by Radke et al. 2020)

Reference	Exposure	Outcome	Selection	Confound	Analysis	Overall
Gascon et al. (2015)*	G/A	G	G	G	G	High
Lien et al. (2015)*, Huang et al. (2019)	A/D	A	D	A	G	Medium
Percy et al. (2016), Braun et al. (2017), Li et al. (2020)	G/A	G	A	G	G	High
Ipapo et al. (2017), Balalian et al. (2019)*, Daniel et al. (2020), Daniel et al. (2020b)	A/D	A	A	G	G	Medium
Doherty et al. (2017)*	A/D	A	A	A	A	Medium
Kim et al. (2018)*	D	D	D	A	A	Low
Olesen et al. (2018)	A/D	A	G	A	A	Medium
Hyland et al. (2019)	G/A	G	A	G	G	High
Qian et al. (2019)	G/A	A	A	A	A	Medium
England – Mason et al. (2020)	A/D	G	A	A	G	Medium
Oulhote et al. (2020)	A/D	A	G	A	G	Medium

G-good, A-adequate, D-deficient, G/A-good for short chain phthalates, adequate for long chain phthalates, D/A-adequate for short chain phthalates, deficient for long chain phthalates

*based on Radke et al. (2020) evaluation

Radke, E.G.; Braun, J.M.; Nachman, R.M.; Cooper, G.S. Phthalate exposure and neurodevelopment: A systematic review and meta-analysis of human epidemiological evidence. *Environ Int.* **2020**, 137, 105408. doi: 10.1016/j.envint.2019.105408.
